# 非小细胞肺癌局部治疗ctDNA-MRD监测研究进展

**DOI:** 10.3779/j.issn.1009-3419.2026.102.11

**Published:** 2026-04-20

**Authors:** Zeyu LI, Yufei WANG, Yongkun WU, Zhanlin GUO

**Affiliations:** ^1^010030 呼和浩特，内蒙古医科大学第一临床医学院; ^1^The First Clinical Medical College, Inner Mongolia Medical University, Hohhot 010030, China; ^2^010050 呼和浩特，内蒙古医科大学附属医院胸外科; ^2^Department of Thoracic Surgery, The Affiliated Hospital of Inner Mongolia Medical University, Hohhot 010050, China

**Keywords:** 肺肿瘤, 循环肿瘤DNA, 分子残留病灶, 局部治疗, 预后, Lung neoplasms, Circulating tumor DNA, Molecular residual disease, Local treatment, Prognosis

## Abstract

非小细胞肺癌（non-small cell lung cancer, NSCLC）局部治疗后仍存在复发风险，如何在影像学进展前识别分子残留病灶并进行风险分层，是当前临床研究的重要问题。基于循环肿瘤DNA（circulating tumor DNA, ctDNA）的分子残留病灶（molecular residual disease, MRD）监测为复发风险评估、疗效判断和后续治疗分层提供了新的分子学工具。然而，手术、热消融、根治性放化疗及立体定向体部放疗后的肿瘤负荷变化、组织损伤和DNA释放与清除过程并不相同，导致ctDNA-MRD采样时点、结果解释及临床应用价值尚未统一。本文旨在综述NSCLC不同局部治疗后ctDNA-MRD首次评估窗口的临床证据，分析治疗方式、检测平台和连续监测对结果判读的影响，并探讨差异化MRD监测策略的临床转化价值。现有证据提示，围手术期ctDNA-MRD状态与病理缓解、复发风险及生存结局有关联；相比之下，热消融和立体定向体部放疗后ctDNA-MRD监测的直接研究证据仍有限，相关采样时间更适合视为候选评估窗口而非标准化推荐。未来仍需通过标准化采样、平台验证及前瞻性干预研究，明确ctDNA-MRD在辅助治疗选择、随访强化和个体化管理中的临床价值。

## Abstract (en)

Non-small cell lung cancer (NSCLC) remains at risk of recurrence after local treatment, and early identification of molecular residual disease before radiological progression is an important issue in risk stratification. Circulating tumor DNA (ctDNA)-based molecular residual disease (MRD) monitoring provides a promising molecular tool for recurrence risk assessment, response evaluation, and subsequent treatment stratification. However, surgery, thermal ablation, definitive chemoradiotherapy, and stereotactic body radiotherapy differ in tumor burden changes, tissue injury, and DNA release and clearance, leading to uncertainty regarding sampling time points, result interpretation, and clinical utility. This review summarizes recent advances in ctDNA-MRD monitoring after local treatment for NSCLC, focusing on ctDNA kinetics, candidate assessment windows, assay platform differences, prognostic value, and translational limitations in perioperative, thermal ablation, and radiotherapy settings. Current evidence suggests that perioperative ctDNA-MRD status is associated with pathological response, recurrence risk, and survival outcomes. In contrast, direct evidence after thermal ablation and stereotactic body radiotherapy remains limited, and proposed sampling time points should be regarded as candidate assessment windows rather than standardized recommendations. Further studies with standardized sampling, assay validation, and prospective interventional designs are needed to clarify the clinical value of ctDNA-MRD in adjuvant treatment selection, intensified surveillance, and individualized management.

肺癌是全球发病率和死亡率最高的恶性肿瘤。全球癌症统计数据^[1]^显示，2022年全球新发肺癌约248万例，肺癌相关死亡约181万例，居所有癌症死因首位。中国国家癌症中心数据^[2]^同样显示，肺癌在我国新发恶性肿瘤和肿瘤相关死亡中均居前列。非小细胞肺癌（non-small cell lung cancer, NSCLC）是肺癌的主要类型。随着外科手术、围手术期系统治疗、影像引导热消融、根治性放化疗及立体定向体部放疗（stereotactic body radiotherapy, SBRT）等治疗模式不断发展，早期及局部晚期NSCLC患者的局部控制手段逐渐丰富^[3-5]^。然而，部分患者在接受根治性或局部控制性治疗后仍会发生局部复发或远处转移。传统影像学主要识别宏观病灶，对微小残留病灶和早期分子复发的识别存在滞后。因此，如何在影像学进展前识别复发高风险人群，并将其纳入后续随访、辅助治疗或临床研究分层，是NSCLC局部治疗后管理中的重要问题。

循环肿瘤DNA（circulating tumor DNA, ctDNA）是循环游离DNA（cell-free DNA, cfDNA）中来源于肿瘤细胞的组成部分，可携带肿瘤相关突变、拷贝数改变、甲基化及片段组学等分子信息。基于ctDNA的分子残留病灶（molecular residual disease, MRD）监测为NSCLC局部治疗后的复发风险分层、疗效评价和动态随访提供了新的分子学工具^[6-8]^。既往研究^[9-11]^显示，治疗后或随访阶段ctDNA检出与较高复发风险相关，且分子学复发可早于影像学进展出现。围手术期研究^[12-14]^进一步提示，ctDNA动态变化不仅与复发风险相关，也可能与病理完全缓解（pathological complete response, pCR）、主要病理缓解（major pathological response, MPR）、无事件生存（event-free survival, EFS）及总生存（overall survival, OS）等结局存在关联。但不同研究在检测平台、采样时点、治疗背景和终点设置方面存在差异，目前ctDNA-MRD更适合作为风险分层和研究分层工具，其能否可独立指导辅助治疗强化或降阶梯治疗，仍需前瞻性干预研究验证。

不同局部治疗方式后的ctDNA动态变化及结果判读背景并不相同。手术切除可迅速降低主要肿瘤负荷，但治疗前、新辅助治疗后、术后预设时点及随访阶段的ctDNA检测，分别侧重于基线风险评估、分子应答评价、术后残留风险判断和分子复发预警。热消融在原位诱导肿瘤坏死，短期ctDNA变化可能同时受到肿瘤DNA释放、组织损伤及炎症反应影响；根治性放化疗和SBRT后的ctDNA变化则具有一定时间依赖性，且直接证据仍不均衡。因此，局部治疗后的ctDNA-MRD监测不应仅理解为单一“首次采血时间”的选择，而应结合治疗方式、采样时点、检测策略、分子负荷变化及驱动基因状态进行综合判读。基于此，本文拟综述NSCLC局部治疗后ctDNA-MRD监测的研究进展，重点讨论围手术期、热消融及放疗后ctDNA动态变化、候选评估窗口、检测平台差异、预后与预测价值及应用限制，以期为后续研究设计和临床结果解释提供参考。

## 1 ctDNA-MRD监测的结果构成与判读要点

### 1.1 ctDNA-MRD的基本概念与临床含义

MRD是指在接受根治性或局部控制性治疗后，常规影像学及临床检查尚不能明确发现残留病灶时，仍可通过分子检测识别的潜在肿瘤残留状态^[8]^。ctDNA是cfDNA中来源于肿瘤细胞的组成部分，可携带肿瘤相关突变、拷贝数改变、甲基化或片段组学特征，为NSCLC局部治疗后的分子复发预警和复发风险评估提供了新的检测手段^[15]^。

需要强调的是，ctDNA-MRD主要反映显微或分子层面的残留风险，其临床含义不同于病理学对切缘状态、残留肿瘤状态及淋巴结转移的评价。切缘状态和残留肿瘤描述仍是评价局部切除完整性的重要病理学依据^[16]^。MRD阳性通常提示复发风险升高，但不能替代病理学和影像学评价，而MRD阴性提示风险相对较低，但仍需考虑低丰度残留、肿瘤低脱落特征、检测灵敏度及采样时点等因素^[8]^。

### 1.2 检测策略与平台差异

ctDNA-MRD检测策略不同，其灵敏度、适用场景和结果解释方式亦存在差异。目前常见策略可概括为肿瘤先验型检测和肿瘤非依赖型检测^[6]^。肿瘤先验型检测通常先对患者肿瘤组织进行测序，筛选个体化突变位点，再在血浆中进行深度追踪。这类方法个体化程度较高，适合已有手术或活检组织标本、需要识别低丰度残留信号的患者。Chen等^[17]^报道的PROPHET个体化肿瘤先验型检测通过追踪患者特异性变异用于早期NSCLC术后MRD监测，显示出较固定基因面板更高的复发风险分层价值。其局限在于依赖肿瘤组织质量和检测周期，同时由于肿瘤存在空间异质性，单一组织取样可能难以覆盖全部肿瘤亚克隆，进而影响后续血浆追踪位点的代表性。

肿瘤非依赖型检测不依赖患者肿瘤组织，而是直接基于血浆样本中的固定基因面板、甲基化特征、片段组学特征或多组学模型识别肿瘤来源信号。Hong等^[18]^采用肿瘤非依赖型甲基化cfDNA检测分析895例可切除早期NSCLC患者术前血浆样本，提示该类方法可在组织信息不足或术前风险评估场景中提供补充性风险分层信息。其局限在于局部治疗后低肿瘤负荷状态下肿瘤来源信号比例较低，检测结果更易受到非肿瘤来源cfDNA、平台阈值、克隆性造血（clonal hematopoiesis, CH）及采样时点等因素影响^[19]^。

### 1.3 MRD结果的分层解读

MRD结果的判读不应局限于“阳性”或“阴性”的定性结论，还应结合分子负荷、变异性质和纵向变化综合分析。分子负荷可通过ctDNA水平、变异等位基因频率（variant allele frequency, VAF）及阳性位点数量等指标体现^[15]^。变异性质主要涉及驱动基因、耐药相关变异及CH干扰的排除^[19]^。纵向变化则包括ctDNA清除、持续阳性、由阴转阳及分子负荷升高等情况，较单次结果更有助于判断复发风险^[20]^。

驱动基因信息是MRD结果判读中的重要组成部分。若ctDNA中检出表皮生长因子受体（epidermal growth factor receptor, EGFR）、间变性淋巴瘤激酶（anaplastic lymphoma kinase, ALK）等具有治疗指向性的分子异常，可能为辅助靶向治疗选择、复发后治疗策略或临床试验入组提供参考^[4]^。但现阶段，单纯依据MRD阳性或某一驱动基因阳性直接改变辅助治疗方案，仍缺乏充分的前瞻性干预证据。因此，MRD的分子层面信息更适合作为风险分层和研究分层依据，而非孤立的治疗决策标准^[6]^。

### 1.4 采样时点与临床背景

ctDNA-MRD结果的临床含义与采样时点密切相关，不同采样阶段对应的临床解释目的并不相同。治疗前样本主要反映基线肿瘤负荷、ctDNA脱落能力及分子特征，可为后续疗效评价和动态比较提供参照。治疗后预设时点样本更多用于评估潜在残留风险，随访阶段样本则有助于识别分子复发和动态风险变化。因此，ctDNA-MRD结果应置于采样时间、治疗暴露、检测平台和分子负荷变化中综合解释，而不宜仅依据某一单次检测进行判断^[8]^。

## 2 围手术期ctDNA-MRD监测

### 2.1 治疗前ctDNA与基线风险评估

围手术期ctDNA-MRD监测不应仅限于术后样本。治疗前ctDNA状态可为后续治疗反应和术后MRD判读提供基线参照，也可能反映肿瘤负荷、DNA脱落特征及生物学侵袭性。LUNGCA-1研究^[21]^显示，术前ctDNA阳性与较差无复发生存（recurrence-free survival, RFS）相关，提示治疗前ctDNA状态具有一定基线风险分层价值。TRACERx研究^[22]^进一步提示，术前未检出ctDNA的部分肺腺癌患者可能具有相对惰性的生物学特征和较好临床结局。因此，治疗前ctDNA检测的意义主要在于建立分子基线和辅助识别复发高风险人群，而不宜被孤立用于判断后续治疗策略。

对于接受新辅助治疗的患者，治疗前ctDNA样本主要用于建立分子基线，并为后续分子应答评价提供参照。

### 2.2 新辅助治疗后ctDNA动态与病理缓解

在新辅助或围手术期治疗模式下，ctDNA动态变化逐渐被用于补充评价治疗反应。NADIM研究^[13]^的长期随访及生物标志物分析提示，治疗前低ctDNA水平以及新辅助治疗后ctDNA不可检出均与较好的无进展生存期（progression-free survival, PFS）和OS相关，且治疗后ctDNA对OS的预测能力优于基于实体瘤疗效评价标准（Response Evaluation Criteria in Solid Tumors, RECIST）的影像学评价。Yue等^[23]^在可切除NSCLC新辅助免疫治疗研究中也观察到，围手术期ctDNA动态变化有助于预测治疗反应和复发风险。上述研究提示，ctDNA清除或下降可作为病理反应之外的补充性疗效评价指标，但其临床解释仍需结合治疗方案、采样时点和检测平台。

ctDNA清除与pCR、MPR分别反映外周血分子信号变化和切除标本中的病理反应，二者不宜简单等同。CheckMate 816研究^[12]^显示，新辅助纳武利尤单抗联合化疗较单纯化疗可显著提高pCR率并延长EFS，为围手术期治疗背景下讨论ctDNA动态和病理反应提供了重要临床基础。基于CheckMate 816的病理反应分析进一步显示，残余活肿瘤（residual viable tumor, RVT）比例与EFS呈梯度相关，RVT比例越低，患者2年EFS结局越好；同时，与影像学反应和ctDNA清除相比，病理反应对EFS的拟合更接近^[14]^。因此，在接受新辅助治疗的NSCLC患者中，ctDNA动态变化更适合作为pCR/MPR的补充指标，而不是替代指标。

### 2.3 术后预设时点MRD与早期检测干扰

术后预设时点MRD检测是围手术期ctDNA研究中证据较为集中的部分。LUNGCA-1研究^[21]^纳入可手术I-III期NSCLC患者，并设置术前、术后3 d及术后1个月等采样点，结果显示术后3 d和/或术后1个月ctDNA阳性定义的MRD状态与疾病复发风险升高相关。LUCID研究^[10]^则显示，术后1-3 d ctDNA检出与复发无关，而治疗后2周至4个月landmark窗口ctDNA检出与较短RFS和OS相关。上述结果提示，术后预设时点MRD阳性具有复发风险分层价值，但术后极早期ctDNA结果仍需结合采样时点、检测平台和后续复测综合解释。

围手术期cfDNA动态是术后早期结果判读中需要考虑的重要因素。Henriksen等^[24]^在结直肠癌和膀胱癌患者中观察到，手术创伤可引起cfDNA水平升高，并可持续数周，从而可能影响低丰度ctDNA信号识别；这一结果为术后极早期ctDNA判读提供了跨癌种的间接证据。Zhao等^[25]^在早期NSCLC围手术期研究中也观察到，cfDNA和ctDNA动态变化可能共同反映术后复发风险。基于上述证据，术后极早期ctDNA结果更适合作为后续复测和随访解释的参考，阴性结果亦不宜单独作为排除MRD的依据。与单次早期检测相比，预设时点复测结合纵向监测更有助于提高复发风险评估的可靠性。

### 2.4 纵向监测与分子复发预警

术后ctDNA-MRD监测的临床价值不仅在于某一固定时点的阳性或阴性结果，更在于通过纵向检测动态评估复发风险。TRACERx研究^[22]^采用个体化多突变追踪策略，在197例NSCLC患者的1069份血浆样本中追踪术后ctDNA变化，提示ctDNA监测可用于观察早期肺癌转移播散和分子复发过程。另一项独立的LUCID研究^[10]^采用个体化RaDaR检测分析88例早期NSCLC患者的连续血浆样本，显示治疗后ctDNA检出与复发风险升高相关，并可早于临床复发出现。上述研究从不同队列提示，纵向ctDNA监测有助于复发风险识别和分子复发预警。

纵向MRD监测还有助于识别复发风险较低的人群。Zhang等^[20]^研究提示，局限期NSCLC患者若在连续随访中保持MRD未检出，可能代表相对低复发风险人群。该队列的后续随访分析进一步显示，预设时点MRD和纵向MRD均具有较高复发预测价值，持续MRD未检出有助于识别潜在低复发风险人群；但MRD阴性结果仍可能受复发部位、肿瘤脱落特征及采样间隔影响，不能直接等同于治愈^[26]^。近期LUNGCA研究^[11]^进一步从全程术后管理角度评估动态ctDNA监测价值，提示综合治疗后MRD状态较单纯术后MRD状态对复发预测更优，支持将ctDNA-MRD理解为贯穿手术、辅助治疗和随访阶段的动态监测工具。

综上，围手术期研究已较一致地提示，治疗前ctDNA阳性、术后或随访阶段MRD阳性、MRD由阴转阳、持续阳性以及分子负荷升高，均与较高复发风险或较差生存结局有关，持续MRD未检出则更多支持相对低风险分层。然而，不同研究在检测平台、采样时间、治疗方案和终点设置方面仍存在差异，现阶段尚不宜将单次ctDNA-MRD结果作为辅助治疗强化、降阶梯治疗或降低随访强度的独立依据。

## 3 非手术局部治疗后ctDNA-MRD监测

### 3.1 热消融后ctDNA-MRD监测

热消融主要包括射频消融和微波消融等方式，常用于部分不能耐受手术或拒绝手术的早期NSCLC患者^[3]^。与手术直接移除肿瘤组织不同，热消融是在原位破坏肿瘤病灶，治疗后短期ctDNA变化可能同时受到肿瘤坏死释放、局部炎症反应、消融范围及非肿瘤来源cfDNA变化等因素影响。cfDNA生物学研究^[27]^提示，cfDNA水平受细胞死亡、主动释放及清除过程共同影响，治疗相关组织损伤和炎症反应均可能改变外周血cfDNA背景。因此，热消融后极早期ctDNA升高不宜直接理解为稳定MRD阳性，ctDNA下降或未检出也不能完全排除局部残留。

目前NSCLC热消融后ctDNA-MRD监测的直接证据仍有限。Cheng等^[28]^在接受微波消融治疗的I期NSCLC患者中，于消融前24 h、消融后1 h内及消融后1-3个月采集血样，发现消融后1 h内ctDNA阳性率由41.9%升至60.7%，提示治疗后短时间内可出现ctDNA释放增加。该研究还显示，消融后1-3个月ctDNA阳性者1年PFS率低于阴性者，且ctDNA较影像学提前提示复发，中位提前时间为4.9个月^[28]^。因此，消融后1-3个月可作为当前证据下较具解释价值的ctDNA-MRD候选评估窗口，这一判断主要来自Cheng等^[28]^研究中的ctDNA随访结果，并与消融后影像学随访中建立治疗后比较基线的临床逻辑相衔接^[28,29]^。

现阶段，热消融后ctDNA-MRD更适合作为影像学随访的补充工具，而不宜单独作为判断局部残留或追加治疗的依据。若ctDNA持续阳性、由阴转阳或分子负荷逐渐升高，应重点复核消融区变化及远处复发风险。已有相关文献^[30]^指出，热消融相关生物标志物研究仍受样本量、观察时点和研究异质性限制，未来需更多前瞻性研究验证。

### 3.2 根治性放疗或放化疗后ctDNA-MRD监测

对于不可手术或局部晚期NSCLC患者，根治性放疗、同步放化疗及后续巩固免疫治疗是重要治疗模式。放疗可通过DNA损伤、细胞凋亡、坏死及延迟性有丝分裂灾难等过程影响肿瘤细胞死亡和DNA释放，其ctDNA动态不同于手术后的快速下降，也不同于热消融后的短期释放。治疗后ctDNA水平受肿瘤细胞死亡、DNA释放、清除效率及背景cfDNA水平等多种因素影响^[31]^。

根治性放疗或放化疗后ctDNA动态变化已显示出一定预后价值。Pan等^[32]^对局部晚期NSCLC患者放化疗过程中的ctDNA进行纵向分析，发现ctDNA浓度在放疗过程中及放疗结束时较基线下降；治疗过程中或治疗结束时ctDNA转为未检出的患者，较ctDNA清除延迟者具有更好的PFS。Lebow等^[33]^采用肿瘤先验型检测评估I-III期NSCLC患者根治性放疗后的MRD状态，发现放疗后ctDNA阳性与较差PFS相关，且ctDNA提示复发可早于影像学进展。这些研究支持根治性放疗或放化疗后ctDNA-MRD用于复发风险分层和分子复发预警。

不过，根治性放疗或放化疗后的首次评估时间仍受检测平台和采样设计影响。肿瘤非依赖型检测研究^[34,35]^显示，在部分接受根治性放化疗的患者中，治疗后较晚时点ctDNA阳性与复发风险及生存结局的关系更为清楚，而较早时点结果的稳定性相对不足。因此，根治性放疗或放化疗后的ctDNA-MRD评估不宜简单归纳为某一统一采样窗口，而应结合检测平台、治疗结束后的具体时间、连续监测结果及影像学随访进行综合判断。Moding等^[36]^进一步提示，放化疗后的ctDNA动态变化可能有助于识别更可能从巩固免疫治疗中获益的患者；但现阶段，这类证据更适合作为风险分层和临床研究设计依据，而非直接替代影像学评估或单独决定巩固治疗策略。

### 3.3 SBRT后ctDNA-MRD监测

SBRT是早期NSCLC的重要非手术局部治疗方式，治疗后可出现磨玻璃影、实变、放射性肺炎及后期纤维化等影像学改变，早期判断局部残留或复发存在一定困难^[29,37]^。因此，ctDNA-MRD监测在SBRT后的潜在价值主要体现在补充影像学判读和辅助识别复发高风险患者。

目前SBRT后ctDNA-MRD监测的直接证据仍有限。SABR-DETECT研究^[38]^显示，首次分次放疗后24-72 h ctDNA检出率可由22.7%升至27.3%，其中部分患者由阴性转为阳性。这提示SBRT后极早期ctDNA变化可能反映治疗诱导的短期DNA释放，而不一定代表稳定MRD状态。另有前瞻性研究^[39]^提示，根治性低分割放疗后早期ctDNA动态峰值可能与后续疾病控制相关，但研究纳入场景并非纯SBRT，样本量也较有限。因此，SBRT后极早期ctDNA变化既可能包含放射生物学响应信息，也可能受到治疗诱导释放和背景波动影响，尚不足以直接确定SBRT后的MRD评估基线。

在当前直接分子证据不足的情况下，SBRT后的ctDNA-MRD候选评估窗口仍需与影像学随访逻辑相衔接。肺癌非手术治疗后影像随访综述指出，治疗后约3个月常用于避开急性放射性肺损伤并建立相对稳定的影像学比较基线^[29]^。据此，SBRT后约3个月可作为当前临床实践中较为稳妥的ctDNA-MRD候选评估时点；但这一时点更多来自影像随访可行性和临床实施逻辑，尚缺乏足够分子动力学证据直接支持。

### 3.4 非手术局部治疗后的结果解释

总体来看，非手术局部治疗后的ctDNA-MRD监测仍处于证据积累阶段。热消融、根治性放疗或放化疗和SBRT后的ctDNA动态并不相同^[28,32,38]^，而非手术局部治疗后的影像学改变也可能影响疗效和复发判断^[29]^。热消融后主要问题在于原位肿瘤坏死及消融区影像变化对早期ctDNA解释的影响；而根治性放疗或放化疗后更强调ctDNA清除速度、残留ctDNA和后续复发或生存结局之间的关系；SBRT后则需要重点关注低肿瘤负荷、低脱落特征和放射后影像学改变带来的判读不确定性。

因此，非手术局部治疗后的ctDNA-MRD结果不宜简单套用术后MRD监测经验，也不宜将某一固定时间点定义为普遍适用的标准采样窗口。现阶段，ctDNA动态变化更适合作为影像学随访和风险分层的补充依据，而非单次检测即可触发治疗调整的独立标准。未来仍需在不同非手术治疗场景下开展标准化采样和前瞻性验证研究，以明确候选评估窗口、阈值标准及临床转化价值。

## 4 临床转化价值与应用限制

ctDNA-MRD监测在NSCLC局部治疗后的临床转化价值，主要体现在复发风险分层、动态随访和临床研究分层。现有证据较一致地提示，治疗后MRD阳性、随访中MRD转阳或分子负荷升高与较高复发风险相关，持续MRD未检出者总体复发风险较低。近期系统评价和meta分析^[40]^显示，在可切除NSCLC中，术后ctDNA检出、MRD评估及纵向ctDNA监测均与较差RFS相关，其中纵向ctDNA监测与复发风险的关联更为显著。Qiu等^[41]^研究进一步提示，术后及辅助治疗后ctDNA阳性均与较差RFS相关，且术后ctDNA状态可能有助于预测辅助化疗获益。因此，ctDNA-MRD可作为病理分期、影像学随访和临床高危因素之外的补充指标，用于识别需要重点随访或适合进入临床研究的人群。

围手术期研究已提示ctDNA状态与病理缓解、复发风险和生存结局相关，根治性放化疗后ctDNA动态变化也可能帮助识别不同预后及潜在治疗获益人群。然而，不同研究在检测平台、采样时点、治疗方案、MRD阳性阈值和终点设置方面存在差异，热消融和SBRT后的直接证据仍有限。因此，现阶段ctDNA-MRD更适合作为风险分层和研究分层工具，而非独立改变辅助治疗、巩固治疗或局部补救治疗的决策标准^[6,8]^。

前瞻性干预研究是ctDNA-MRD由“预后标志物”走向“决策工具”的关键。MERMAID-1研究^[42]^以完全切除II-III期NSCLC术后MRD阳性患者为主要目标人群，评估度伐利尤单抗联合标准辅助化疗相较安慰剂联合标准辅助化疗的疗效，体现了MRD阳性人群辅助治疗强化的验证思路。MERMAID-2研究^[43]^则将根治性治疗后随访期间MRD转阳作为随机化触发条件，纳入影像学尚无复发证据的II-III期NSCLC患者，评估度伐利尤单抗能否延缓疾病复发。这类研究的意义不在于证明MRD检测已经可以常规决定治疗，而在于检验“基于MRD状态进行治疗干预”是否真正带来临床获益。未来研究还需进一步明确不同平台的检测性能、MRD阳性阈值、动态转阳定义、采样流程和影像学随访整合方式。

驱动基因状态是ctDNA-MRD临床转化中需要重点关注的另一维度。对于EGFR突变等具有明确治疗指向性的NSCLC患者，MRD状态可能为辅助靶向治疗时长、停药后复发风险及后续监测策略提供补充信息。ADAURA研究的探索性MRD分析^[44]^显示，在接受辅助奥希替尼治疗的可切除EGFR突变NSCLC患者中，基于血浆的肿瘤先验型MRD分析可用于预测治疗期间及治疗后的复发风险。同时，已有研究方案开始探索以ctDNA-MRD状态指导EGFR突变早期NSCLC患者辅助奥希替尼治疗，提示驱动基因阳性人群的MRD指导治疗正进入前瞻性验证阶段^[45]^。不过，这些证据目前仍不能支持单纯依据MRD阳性或阴性来常规决定辅助靶向治疗强度或疗程。我国NSCLC MRD专家共识也强调，MRD检测的临床应用仍需围绕定义、检测技术、采样流程、结果报告和干预研究进行标准化^[46]^。因此，ctDNA-MRD未来的临床转化应建立在标准化检测、纵向监测和前瞻性干预证据共同支持的基础上。

## 5 总结与展望

ctDNA-MRD监测为NSCLC局部治疗后的复发风险识别和动态随访提供了新的分子学工具。与传统影像学主要识别宏观病灶不同，ctDNA-MRD可从外周血层面反映潜在肿瘤残留和分子复发风险。然而，不同局部治疗方式后的肿瘤负荷变化、DNA释放与清除过程、组织损伤背景及检测平台特征并不相同。因此，ctDNA-MRD结果不能脱离治疗方式和采样时点进行简单解释。现有证据相对支持围手术期和根治性放疗或放化疗后的ctDNA-MRD用于复发风险分层和分子复发预警，而热消融和SBRT后的直接证据仍较有限，相关采样时点更适合视为候选评估窗口，而非标准化推荐。

总体而言，NSCLC局部治疗后ctDNA-MRD监测的价值不在于寻找适用于所有患者和所有治疗方式的统一采血时间点，而在于基于治疗背景、检测平台和连续监测结果建立差异化判读框架。未来仍需通过标准化采样流程、检测平台性能验证、MRD阳性阈值统一及前瞻性干预研究，进一步明确ctDNA-MRD能否真正改善辅助治疗选择、巩固治疗分层、局部补救治疗决策和随访管理策略。随着证据不断积累，ctDNA-MRD有望从复发风险评估工具逐步发展为NSCLC局部治疗后个体化管理的重要组成部分。
